# Anti-CRISPR Phages Cooperate to Overcome CRISPR-Cas Immunity

**DOI:** 10.1016/j.cell.2018.05.058

**Published:** 2018-08-09

**Authors:** Mariann Landsberger, Sylvain Gandon, Sean Meaden, Clare Rollie, Anne Chevallereau, Hélène Chabas, Angus Buckling, Edze R. Westra, Stineke van Houte

**Affiliations:** 1 ESI and CEC, Biosciences, University of Exeter, Cornwall Campus, Penryn TR10 9EZ, UK; 2 CEFE UMR 5175, CNRS Université de Montpellier Université Paul-Valéry Montpellier EPHE, 34293 Montpellier Cedex 5, France

**Keywords:** CRISPR-Cas, bacteria, phage, partial resistance, immunosuppression, anti-CRISPR, epidemiology, tipping points, bifurcation, Allee effect

## Abstract

Some phages encode anti-CRISPR (*acr*) genes, which antagonize bacterial CRISPR-Cas immune systems by binding components of its machinery, but it is less clear how deployment of these *acr* genes impacts phage replication and epidemiology. Here, we demonstrate that bacteria with CRISPR-Cas resistance are still partially immune to Acr-encoding phage. As a consequence, Acr-phages often need to cooperate in order to overcome CRISPR resistance, with a first phage blocking the host CRISPR-Cas immune system to allow a second Acr-phage to successfully replicate. This cooperation leads to epidemiological tipping points in which the initial density of Acr-phage tips the balance from phage extinction to a phage epidemic. Furthermore, both higher levels of CRISPR-Cas immunity and weaker Acr activities shift the tipping points toward higher initial phage densities. Collectively, these data help elucidate how interactions between phage-encoded immune suppressors and the CRISPR systems they target shape bacteria-phage population dynamics.

## Highlights

• Bacteria with CRISPR immunity remain partially resistant to Acr-phage
• Sequentially infecting Acr-phages cooperate to overcome CRISPR resistance
• Acr-phage epidemiology depends on the initial phage density
• CRISPR-resistant bacteria can drive Acr-phages extinct

## Graphical Abstract

**Figure:**
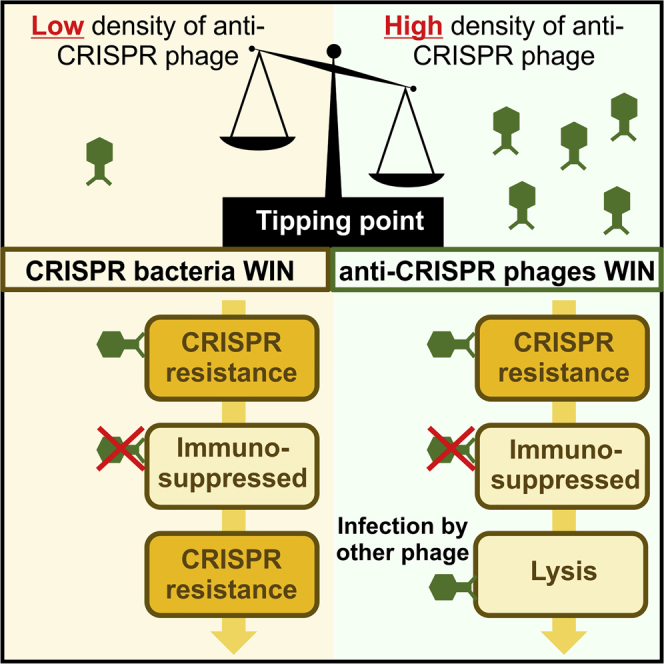

## Teaser

Phage anti-CRISPR proteins weaken the host bacterium’s initial immune response, leading to immunosuppression and leaving the host vulnerable to future phage infection.

## Introduction

Bacteriophages (phages) are highly abundant in virtually all environments and are thought to play a key role in shaping the ecology and evolution of their bacterial hosts (Koskella and Brockhurst, 2014, Weitz, 2015, van Houte et al., 2016a). In response to phage predation, many bacteria evolved defense mechanisms that provide protection against phage predation (Labrie et al., 2010), including many with molecular mechanisms that are only starting to be understood (Makarova et al., 2011, Goldfarb et al., 2015, Doron et al., 2018). The observation that phages persist in the environment in spite of these host defenses is likely due to a combination of ecological and evolutionary processes (Thompson, 2005). One factor that is important in this context is the evolution of phage genes that can specifically block host defenses (Samson et al., 2013). For example, approximately half of the sequenced bacterial genomes encode CRISPR-Cas immune systems (Grissa et al., 2007, Makarova et al., 2015), and in response, many phages have evolved anti-CRISPR (*acr*) genes that inhibit the CRISPR immune response (Bondy-Denomy et al., 2013, Pawluk et al., 2014, Pawluk et al., 2016a). However, the conditions and extent to which these immunosuppressive genes allow phages to persist in the face of bacteria with CRISPR resistance have remained unclear.

CRISPR-Cas immune systems provide defense against phage infection by inserting phage-derived sequences (spacers) into CRISPR loci on the host genome (Barrangou et al., 2007). The evolution of CRISPR resistance is determined both by the abundance of phage genome fragments in the cell that serve as substrates for spacer acquisition as well as the relative fitness advantage of bacteria with newly acquired spacers (Levin, 2010, Swarts et al., 2012, Datsenko et al., 2012, Hynes et al., 2014, Levy et al., 2015, Westra et al., 2015, Künne et al., 2016, Semenova et al., 2016, Severinov et al., 2016). Following the acquisition of novel spacers, processed transcripts of CRISPR loci guide CRISPR-associated (Cas) surveillance complexes and effector nucleases to detect and destroy complementary genomes of re-infecting phages (Brouns et al., 2008, Garneau et al., 2010). In some natural populations and experimental systems, phages can coexist for relatively long periods of time with their bacterial hosts despite the evolution of CRISPR resistance (Weinberger et al., 2012, Paez-Espino et al., 2013, Paez-Espino et al., 2015, Levin et al., 2013, Sun et al., 2016;), which may be explained by incomplete immunity provided by some CRISPR-Cas systems (Levin et al., 2013), phage coevolution with CRISPR-resistant hosts (i.e., escaping CRISPR resistance through the evolution of point mutations in the sequences that are targeted on the phage genome) (Deveau et al., 2008, Semenova et al., 2011, Childs et al., 2012, Iranzo et al., 2013), the loss of CRISPR resistance in some bacterial clones in the population (Weissman et al., 2018), or the immigration of sensitive hosts into the CRISPR-resistant population (Chabas et al., 2016, reviewed in Westra et al., 2016). However, in other cases, the evolution of CRISPR resistance causes rapid phage extinctions, particularly if CRISPR-Cas immune systems generate high population-level spacer diversity, which is difficult for the phage to overcome by point mutation (Childs et al., 2014, van Houte et al., 2016b, Morley et al., 2017). In this context, *acr* genes can provide a critical benefit to the phage by antagonizing the CRISPR immune systems of their bacterial hosts (van Houte et al., 2016b).

All Acrs characterized to date function by inhibiting either the CRISPR surveillance complexes or the effector nucleases (Bondy-Denomy et al., 2015, Pawluk et al., 2016b, Pawluk et al., 2017, Wang et al., 2016a, Wang et al., 2016b, Chowdhury et al., 2017, Dong et al., 2017, Guo et al., 2017, Harrington et al., 2017, Hynes et al., 2017, Peng et al., 2017, Rauch et al., 2017, Shin et al., 2017, Yang and Patel, 2017, Hong et al., 2018). These *acr* genes were first identified in temperate *Pseudomonas* phages (Bondy-Denomy et al., 2013) and can rescue phage from CRISPR-mediated extinction (van Houte et al., 2016b). However, previously reported data suggests that their ability to block CRISPR resistance is imperfect and that some Acrs are more potent than others (Bondy-Denomy et al., 2013). For example, phages encoding AcrIF1 had greater levels of infectivity on CRISPR resistant hosts compared to phages encoding AcrIF4, but in all cases, Acr-phage infectivity was highest on hosts lacking CRISPR-Cas immunity (Bondy-Denomy et al., 2013). While these data suggest that CRISPR immunity provides partial resistance against Acr-phage infection, it has remained unclear how these patterns of partial resistance impact the ability of Acr-phages to replicate and amplify. Here, we demonstrate that Acr-phages need to cooperate in order to overcome partial resistance of CRISPR immune hosts. This requirement for cooperation has important epidemiological consequences as it causes Acr-phages to be driven extinct if their initial titers are below a critical threshold value but allows them to amplify when their titers exceed this threshold.

## Results

### CRISPR-Cas Confers Partial Immunity to Acr-Phages

**Figure 1:**
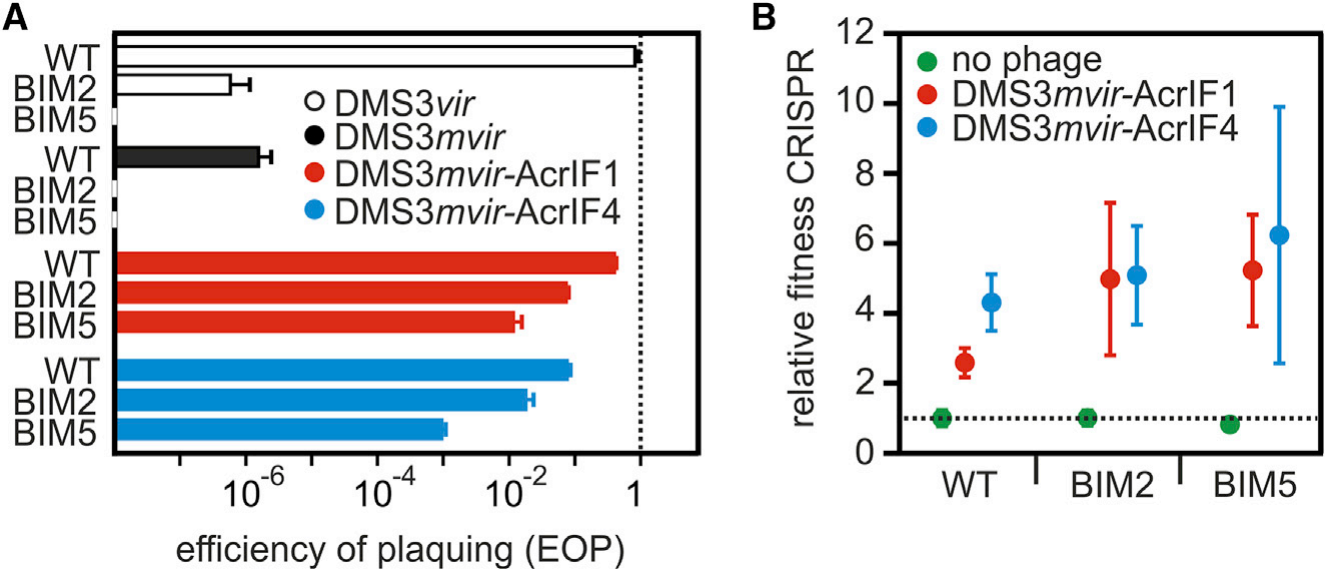
CRISPR-Cas Confers Partial Immunity to Acr-Phages (A) Efficiency of plaquing (EOP) of DMS3*vir* (white bars) on PA14 WT (completely sensitive to DMS3*vir*), BIM2 (one spacer targeting DMS3*vir*), and BIM5 (four spacers targeting DMS3*vir*); EOP of DMS3*mvir* (black bars), DMS3*mvir*-AcrIF1 (red bars), and DMS3*mvir*-AcrIF4 (blue bars) on PA14 WT (one spacer targeting DMS3*mvir*, DMS3*mvir*-AcrIF1, and DMS3*mvir*-AcrIF4), BIM2 (two spacers targeting DMS3*mvir*, DMS3*mvir*-AcrIF1, and DMS3*mvir*-AcrIF4), and BIM5 (five spacers targeting DMS3*mvir*, DMS3*mvir*-AcrIF1, and DMS3*mvir*-AcrIF4). Data correspond to the mean of six independent replicate experiments. Error bars represent 95% confidence intervals (CI). (B) Fitness of bacteria with CRISPR resistance (PA14 WT, BIM2, and BIM5) relative to a phage-sensitive CRISPR-KO strain in the absence of phage (green data points) or in the presence of phage DMS3*mvir*-AcrIF1 (red data points) or phage DMS3*mvir*-AcrIF4 (blue data points). Data correspond to the mean of six independent replicate experiments; error bars represent 95% CI. See also Figure S1.

**Figure S1:**
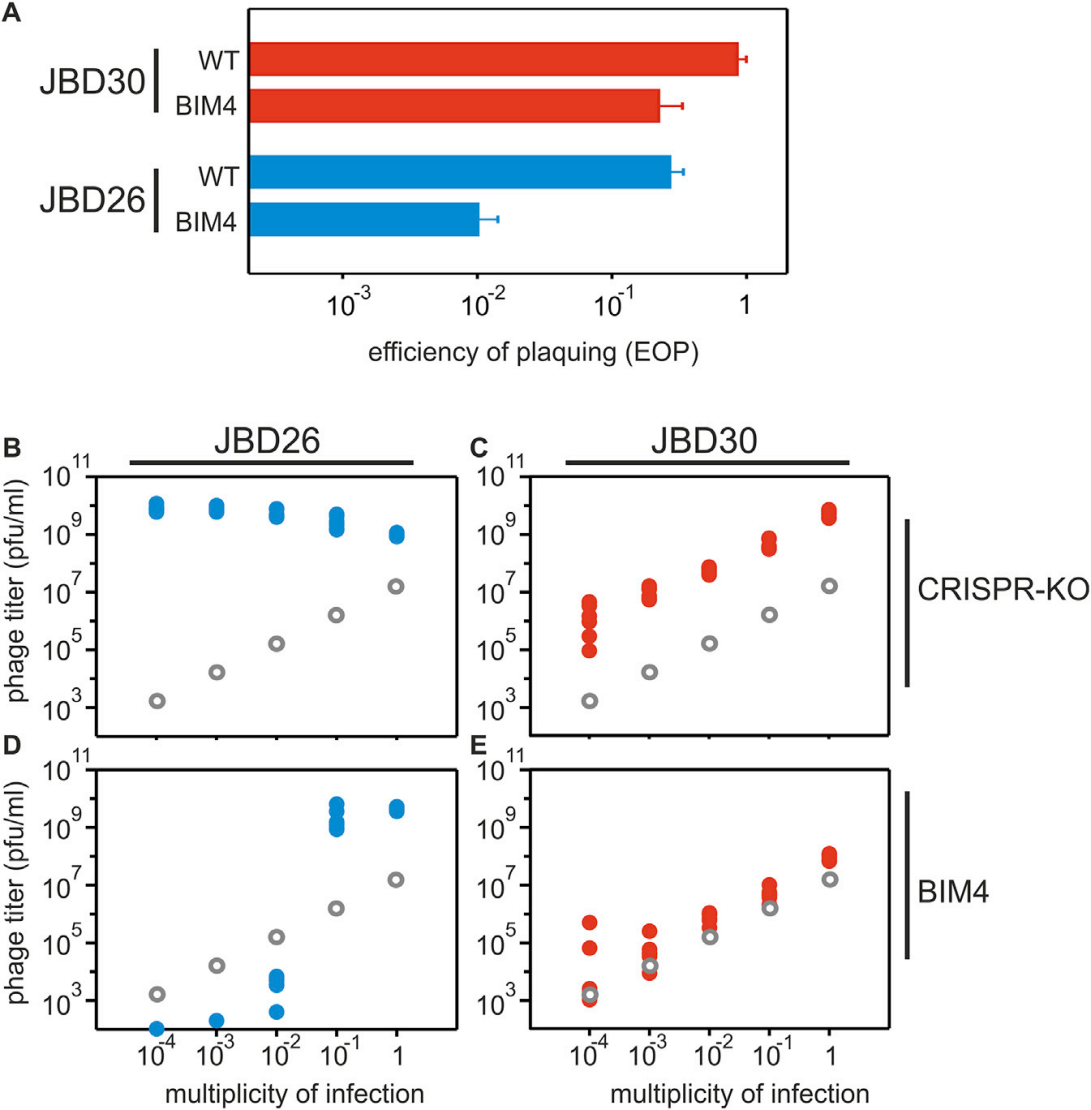
Bacteria with CRISPR Resistance Are Partially Immune to JBD26 and JBD30, Respectively, Related to Figure 1 (A) Efficiency of plaquing (EOP) of JBD26, which encodes AcrIF4 (blue bars) and JBD30, which encodes AcrIF1 (red bars) on BIM4 (1 newly acquired spacer against both phages). Data correspond to the mean of 6 independent replicate experiments. Error bars represent 95% c.i. (B–E) Viral titers at 24 hours post-infection (hpi) with (B) JBD26 and (C) JBD30 on the CRISPR KO strain, (D) JBD26 and (E) JBD30 on BIM4. Gray circles indicate the phage titers (pfu/ml) at the start of the experiment (corresponding to the addition of 10^4^, 10^5^, 10^6^, 10^7^ or 10^8^ pfus). Colored data points represent phage titers at 24 hpi. Note that the observed differences in amplification of JBD30 and JBD26 on CRISPR KO may be due to differences in their rates of lysogenisation. Each experiment was performed as 6 independent replicates, error bars represent 95% c.i.

To investigate the consequences of the partial resistance of CRISPR immune bacteria against Acr-phages (Figure S1A), we expressed AcrIF1 (from phage JBD30) and AcrIF4 (from phage JBD26) in an isogenic phage DMS3*mvir* background, which lacks an endogenous AcrIF but is closely related to both parental phages (91% and 80% pairwise sequence identity, respectively). Consistent with previous observations (Bondy-Denomy et al., 2013), efficiency of plaquing (EOP) assays with DMS3*mvir*-AcrIF1 and DMS3*mvir*-AcrIF4 confirmed partial immunity of *P. aeruginosa* strain UCBPP-PA14 (WT PA14) hosts with CRISPR resistance to these Acr-phages and demonstrated that Acrs differ in their ability to block CRISPR resistance, with AcrIF1 being a more potent suppressor of CRISPR resistance than AcrIF4 (Figure 1A). As expected, EOPs of Acr-phages on wild-type (WT) hosts were higher compared to ancestral phage DMS3*mvir*, which is *a priori* targeted by one spacer of the WT PA14 CRISPR-Cas system but lower than those of phage DMS3*vir*, which is not *a priori* targeted by the WT PA14 CRISPR-Cas system (Figure 1A). Furthermore, EOPs decreased when hosts carried two or five (hereafter named BIM2 and BIM5 [bacteriophage insensitive mutant]) targeting spacers, presumably because this increases the proportion of surveillance complexes that target the phage (in addition to the targeting spacers, all bacteria encode 35 non-targeting spacers). Furthermore, competition between bacteria with CRISPR resistance and sensitive bacteria showed that, in the presence of Acr-phages, CRISPR resistance provides a fitness advantage (Figure 1B; F_1,53_ = 193.98, p < 0.0001), which is consistent with the observation that targeting spacers provide partial resistance to Acr-phages.

### The Initial MOI of Acr-Phages Determines the Epidemiological Outcome

**Figure 2:**
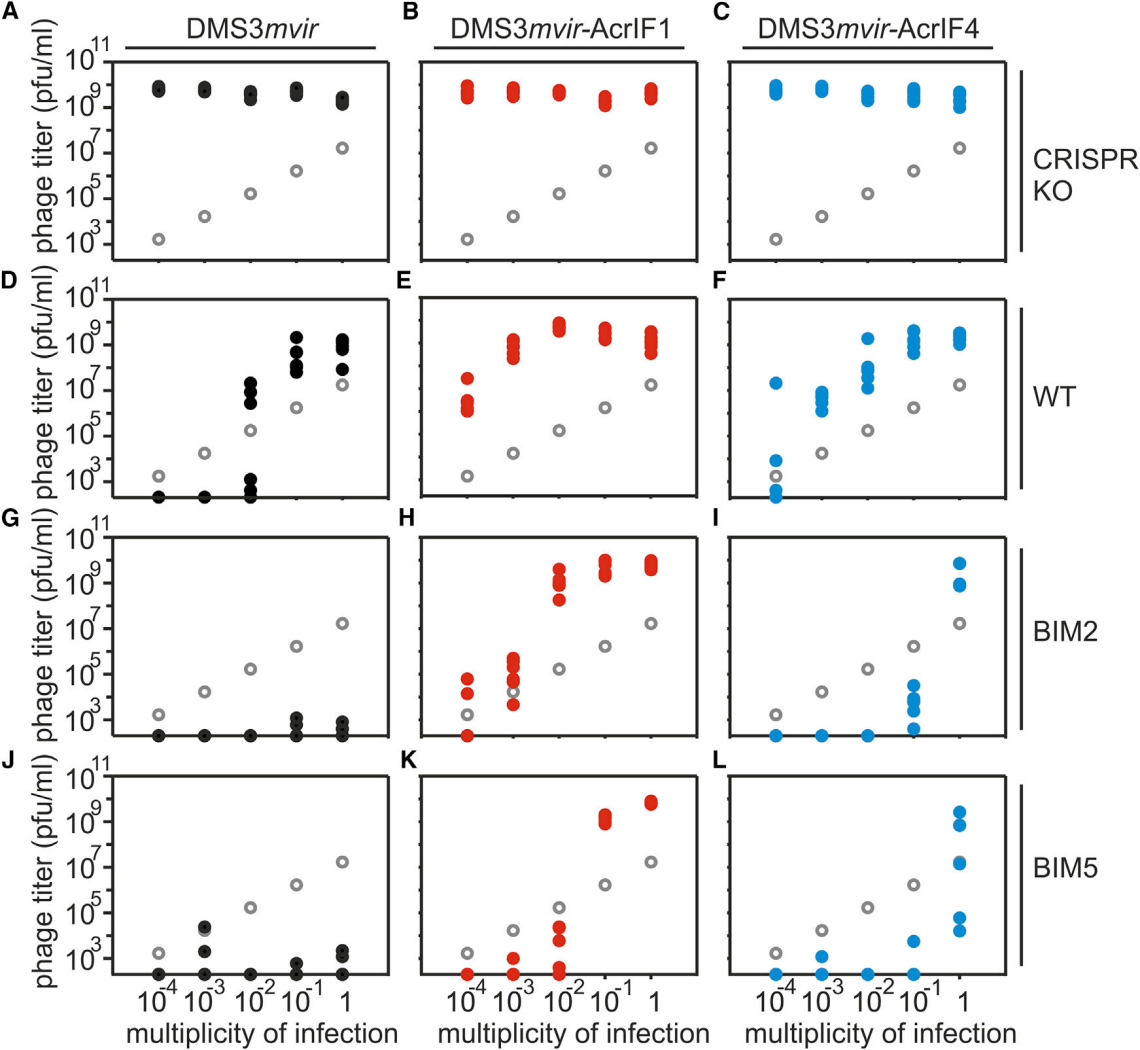
The Initial MOI of Acr-Phage Determines the Epidemiological Outcome (A–L)Viral titers at 24 hr post-infection (hpi) with DMS3*mvir* (A, D, G, and J), DMS3*mvir*-AcrIF1 (B, E, H, and K), or DMS3*mvir*-AcrIF4 (C, F, I, and L) of PA14 CRISPR-KO (A–C), WT (D–F), BIM2 (G–I), or BIM5 (J–L). Gray circles indicate the phage titers at the start of the experiment (corresponding to the addition of 10^4^, 10^5^, 10^6^, 10^7^, or 10^8^ pfus). Colored data points represent phage titers at 24 hpi; each data point represents an independent biological replicate (n = 6). The limit of detection is 200 pfu/ml. See also Figure S2.

While full CRISPR resistance can drive phages extinct (van Houte et al., 2016b), the phage epidemiology associated with partial resistance to Acr-phages is unclear. We explored this by measuring phage amplification following infection of hosts with CRISPR resistance. Whereas phages always reached similar titers when amplified for 24 hr on CRISPR-KO (knockout) hosts, independent of the initial phage amount (Figures 2A–2C), phage amplification on WT bacteria (one targeting spacer) was dependent on the initial phage amount, with phage DMS3*mvir* amplifying exclusively beyond a threshold of around 10^6^ plaque-forming units (pfus), corresponding to an approximate multiplicity of infection (MOI) of 10^−2^ (Figures 2D–2F). For the Acr-phages, this effect was even stronger on BIM2 (two targeting spacers) and BIM5 (five targeting spacers) hosts, revealing epidemiological tipping points that depend both on the level of host resistance and the strength of the Acr (Figures 2G–2L). DMS3*mvir*-AcrIF1 could only cause an epidemic on BIM2 if the initial amount of phage exceeded a threshold of ∼10^5^ pfus, corresponding to an MOI of 10^−3^, and was driven extinct below this threshold (Figure 2H), and for DMS3*mvir*-AcrIF4, approximately 100-fold more phage was necessary to cause an epidemic (Figure 2I). On BIM5, the tipping point shifted to approximately 10-fold higher phage titers for both Acr-phages (Figures 2K and 2L). Similar experiments with the parental phages JBD26 and JBD30 revealed a qualitatively similar correlation between EOPs and phage amplification patterns on bacteria with CRISPR resistance (one newly acquired targeting spacer) (Figure S1), with a relatively low EOP and clear amplification threshold for JBD26 (Figures S1A and S1D) and relatively high EOP and no clear amplification threshold for JBD30 (Figures S1A and S1E). However, unlike the DMS3*mvir*-based isogenic mutants, JBD26 and JBD30 also displayed CRISPR-independent variation in their epidemiological dynamics (Figures S1B and S1C), suggesting that differences in the amplification patterns of JBD26 and JBD30 on CRISPR-resistant hosts are unlikely to be solely due to their different Acrs.

### Epidemiological Tipping Points in Acr-Phage Amplification Are Not Due to Phage Evolution

**Figure S2:**
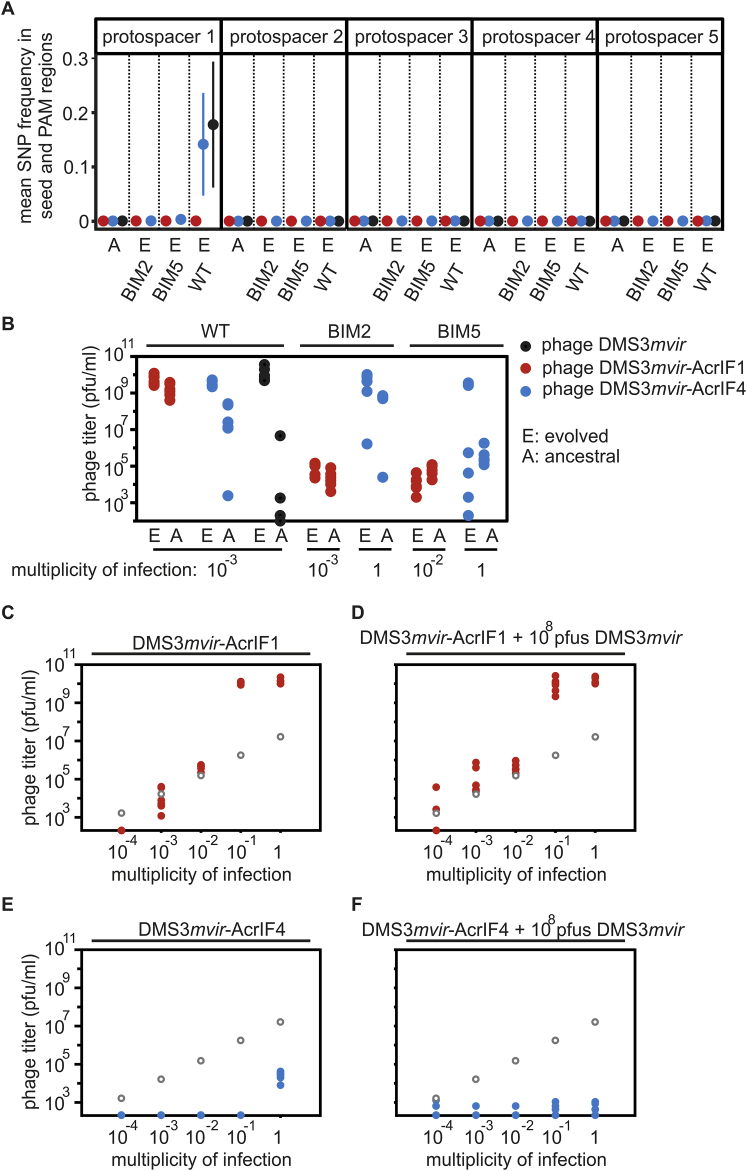
Epidemiological Tipping Points Cannot Be Explained by Phage Evolution or Csy Complex Sequestration, Related to Figure 2 (A) Deep sequencing of protospacer sequences of phages DMS3*mvir* (black data points), DMS3*mvir*-AcrIF1 (red data points) or DMS3*mvir*-AcrIF4 (blue data points), either ancestral (A) or evolved on WT, BIM2 or BIM5 hosts (DMS3*mvir* only on WT). Protospacer 1 is targeted by WT, BIM2 and BIM5, protospacer 2 is targeted by BIM2 and BIM5, and protospacers 3, 4 and 5 are targeted by BIM5. Mean SNP frequency across the seed and PAM region (in total 10 nucleotides) of each protospacer is shown, error bars indicate the 95% c.i. (B) Density-dependent epidemiological tipping points are not due to phage evolution. Viral titers at 24 hpi of phage DMS3*mvir* (black data points), DMS3*mvir*-AcrIF1 (red data points) or DMS3*mvir*-AcrIF4 (blue data points) on bacteria PA14 WT, BIM2 or BIM5. Below each diagram is indicated which phage amounts (pfus) were added in the experiment. A indicates ancestral phage; E indicates evolved phage (isolated from the experiments depicted in Figure 2). (C) Viral titers at 24 hpi of BIM2 with 10^4^, 10^5^, 10^6^, 10^7^ or 10^8^ pfus DMS3*mvir*-AcrIF1. (D) Viral titers at 24 hpi of BIM2 with 10^4^, 10^5^, 10^6^, 10^7^ or 10^8^ pfus DMS3*mvir*-AcrIF1 in the presence of 10^8^ pfus DMS3*mvir*. (E) Viral titers at 24 hpi of BIM2 with 10^4^, 10^5^, 10^6^, 10^7^ or 10^8^ pfus DMS3*mvir*-AcrIF4. (F) Viral titers at 24 hpi of BIM2 with 10^4^, 10^5^, 10^6^, 10^7^ or 10^8^ pfus DMS3*mvir*-AcrIF4 in the presence of 10^8^ pfus DMS3*mvir*. Grey circles indicate the phage titers (pfu/ml) at the start of the experiment (corresponding to the addition of 10^4^, 10^5^, 10^6^, 10^7^ or 10^8^ pfus). Colored points represent phage titers at 24 hpi; each data point represents an independent biological replicate (n = 6). The limit of detection is 200 pfu/ml.

Given that DMS3*mvir*-AcrIF1 and DMS3*mvir*-AcrIF4 epidemics often required the bacterial cultures to be infected with higher amounts of phages, we hypothesized that they might be caused by rare phage mutants that “escape” (partial) CRISPR resistance due to mutations in their target sequence (protospacer) (Antia et al., 2003, Deveau et al., 2008, Semenova et al., 2011, Levin et al., 2013, van Houte et al., 2016b). To test this, we re-sequenced the genomes of phage populations that were isolated from the observed epidemics on WT, BIM2, and BIM5 following infection with 10^8^ pfus, corresponding to an MOI of 1 (i.e., from Figures 2D–2F, 2H, 2I, 2K, and 2L). This showed that the epidemic caused by control phage DMS3*mvir* on WT bacteria was indeed caused by phage that carried a mutated protospacer (i.e., mutation in the seed and protospacer adjacent motif [PAM] region) (Figure S2A). However, in the context of Acr-phages, we found only one example, namely that of DMS3*mvir*-AcrIF4 on WT bacteria, where the epidemic was associated with a protospacer mutation (Figure S2A). For all other Acr-phage epidemics, protospacer SNP frequencies were similar to those of the ancestral phage (Figure S2A). In these cases, we could also not detect any differences in the ability of evolved and ancestral phages to amplify on the hosts with CRISPR resistance they were isolated from (Figure S2B). Therefore, unless the Acr is weak and the host carries only one spacer, phage evolution cannot explain the observed epidemiological tipping points of Acr-phages.

### Acr-Phage Amplification Is Density Dependent

**Figure 3:**
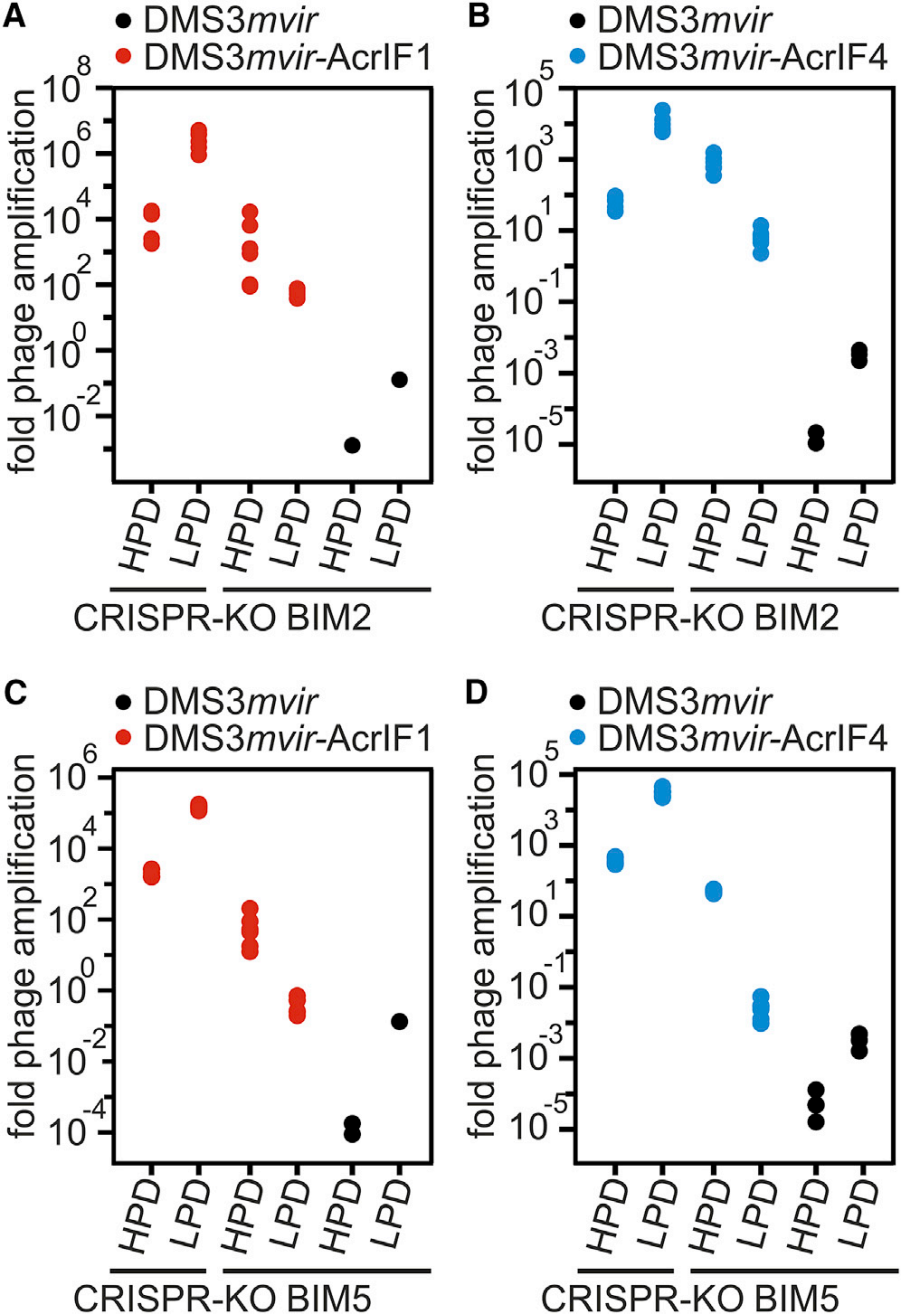
Acr-Phage Amplification Is Density Dependent (A) Fold phage amplification at 24 hpi with 10^6^ pfus DMS3*mvir* (black data points) or DMS3*mvir*-AcrIF1 (red data points) of PA14 CRISPR-KO (sensitive) or BIM2 under either high phage densities (HPD, 6 mL culture) or low phage densities (LPD, 600 mL culture). (B) Fold phage amplification at 24 hpi with 10^8^ pfus DMS3*mvir* (black data points) or DMS3*mvir*-AcrIF4 (blue data points) of PA14 CRISPR-KO (sensitive) or BIM2 under either HPD or LPD. (C) Fold phage amplification at 24 hpi with 10^7^ pfus DMS3*mvir* (black data points) or DMS3*mvir*-AcrIF1 (red data points) of PA14 CRISPR-KO (sensitive) or BIM5 under either HPD or LPD. (D) Fold phage amplification at 24 hpi with 10^8^ pfus DMS3*mvir* (black data points) or DMS3*mvir*-AcrIF4 (blue data points) of PA14 CRISPR-KO (sensitive) or BIM5 under either HPD or LPD; each data point represents an independent biological replicate (n = 6). The limit of detection is 200 pfu/ml. See also Figure S3 and Table S1.

**Figure S3:**
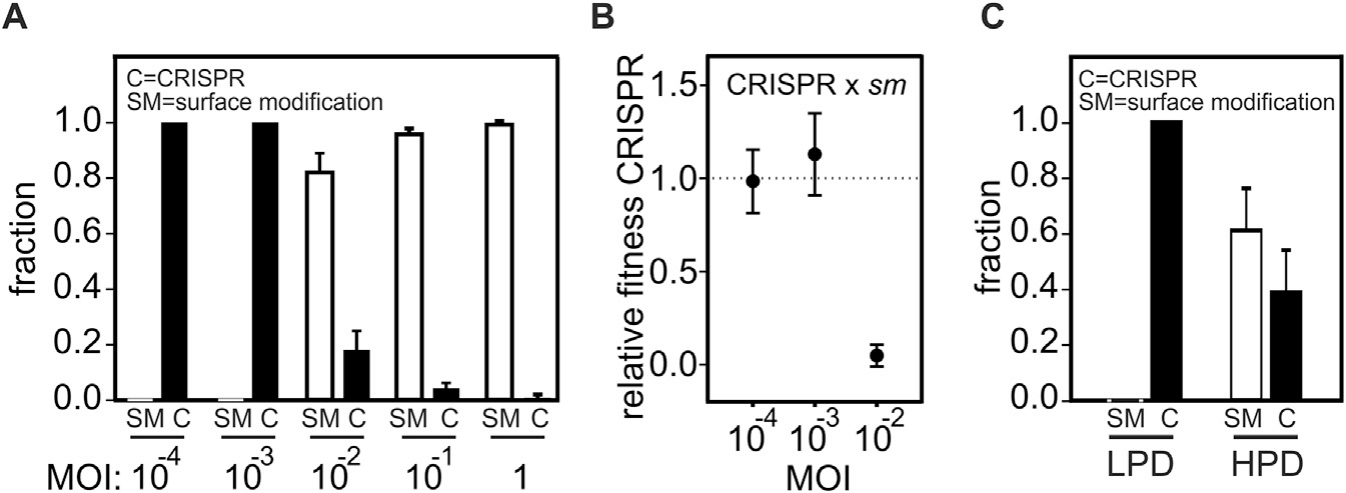
Surface Mutants Invade the Bacterial Population at High Acr-Phage Densities, Related to Figure 3 (A) Resistance phenotypes (C = CRISPR resistance, SM = surface resistance) that evolved at 48 hpi with phage DMS3*vir*-AcrIF1 at the indicated MOIs. All bacteria in the populations initially had CRISPR resistance (2 spacers targeting the phage). Phage amplification was observed at an MOI of 10^−2^ or higher. (B) Relative fitness of bacteria with CRISPR resistance (2 spacers targeting the phage) and surface resistance in the presence of phage DMS3*vir*-AcrIF1 at the indicated MOIs. (C) Resistance phenotypes (C = CRISPR resistance, SM = surface resistance) that evolved at 48 hpi with phage DMS3*vir*-AcrIF1 under either high phage densities (HPD, 6 mL culture) or low phage densities (LPD, 600 mL culture). All bacteria in the populations initially had CRISPR resistance (2 spacers targeting the phage). Data correspond to the mean of 6 independent replicate experiments. Error bars represent 95% c.i.

Having ruled out that the observed tipping points by Acr-phages are the result of escape-phage evolution, we hypothesized that the density of Acr-phages may determine the observed tipping points. To test this hypothesis, we examined whether amplification of Acr-phages was density dependent without altering the initial amount of phages. This was done by measuring amplification of the same initial amount of phages on different volumes of bacterial host culture, generating a high phage-density (HPD) condition (small volume), and a low phage-density (LPD) condition (large volume). Phage amplification was greater on CRISPR-KO hosts under LPD conditions compared to HPD conditions, simply because the bacterial densities are constant across the treatments and the large volume therefore contains proportionally more bacteria on which the phage can replicate (Figure 3; Table S1; F_1,47_ = 64.79, p < 0.0001). However, when Acr-phages were amplified on hosts with CRISPR resistance (BIM2 or BIM5), the greatest level of amplification was observed under HPD conditions (Figure 3; Table S1; F_1,47_ = 47.17, p < 0.0001), demonstrating that Acr-phage amplification is indeed positively density dependent. Furthermore, the level of amplification of Acr-phages on a host with CRISPR resistance was independent of the presence of high amounts of phage DMS3*mvir*, which lacks an *acrIF* gene, demonstrating that the observed density dependence is specifically linked to the density of *acr* genes and cannot be explained by saturation of CRISPR-Cas complexes with targeted phage genomes (Figures S2C–S2F). Consistent with Acr-phages only being able to successfully overcome CRISPR resistance at high phage densities, we observed at 2 days post-infection (dpi) an invasion of bacteria with surface-based resistance under high Acr-phage-density treatments, which was accompanied by a reduction in relative fitness of CRISPR-resistant bacteria (Figure S3; F_1,17_ = 48.9, p < 0001).

### Epidemiological Tipping Points Can Result from Cooperation between Sequentially Infecting Acr-Phages

**Figure 4:**
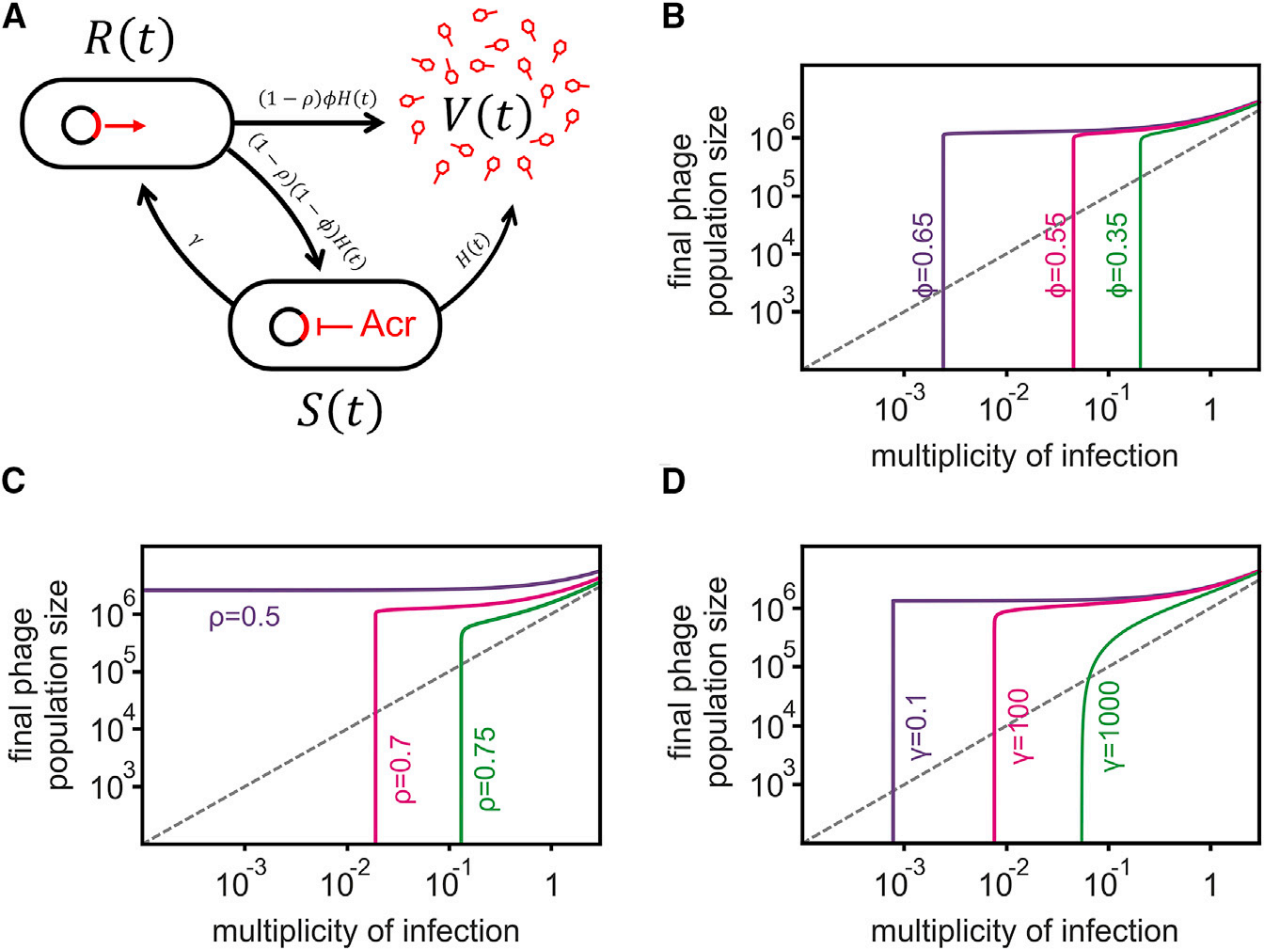
Epidemiological Tipping Points Can Result from Cooperation between Sequentially Infecting Acr-Phages (A) Infection model of the Acr-phage (see details of the model in STAR Methods). The parameter *H(t)* = *αV(t)* refers to the rate at which bacteria are infected by free phage particles. (B) Effect of initial Acr-phage inoculum density on the phage density at 24 hpi for different values of Acr efficacy (purple, ϕ = 0.65; magenta, ϕ = 0.55; green, ϕ = 0.35); other parameter values: *B* = 5, α = 0.001, ρ = 0.7, γ = 20. (C) Effect of initial Acr-phage inoculum density on the phage density at 24 hpi for different values of CRISPR efficacy (ρ = 0.5, 0.7, and 0.75; purple, magenta, green, respectively); other parameter values: *B* = 5, α = 0.001, ϕ = 0.6, γ = 20. (D) Effect of initial Acr-phage inoculum density on the phage density at 24 hpi for different values of the duration of the immunosuppressive state (γ = 0.1, 100, and 1,000; purple, magenta, green, respectively); other parameter values: *B* = 5, α = 0.001, ϕ = 0.65, ρ = 0.7. In all graphs, gray lines correspond to the initial amount of phage, and values below this line indicate a lack of phage amplification. See also Figure S4.

**Figure S4:**
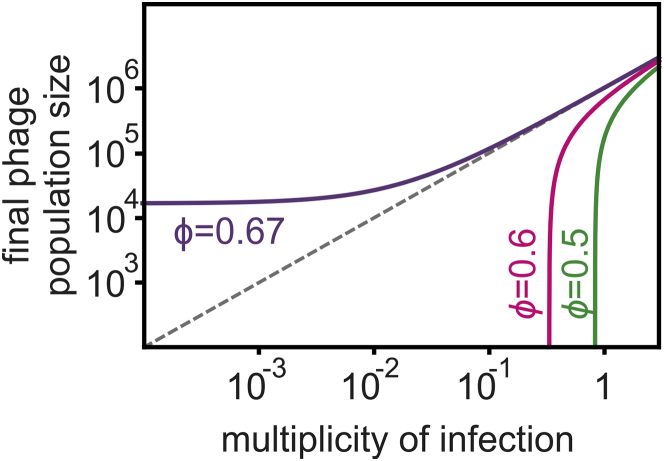
Partial Immunity Alone Cannot Explain the Observed Epidemiological Tipping Points, Related to Figure 4 Model predictions of the effect of initial Acr-phage inoculum density on the phage density at 24hpi for different values of Acr strength when no immunosuppressive state *S* is assumed in the model (ϕ = 0.67, 0.6 and 0.5; purple, magenta, green respectively); other parameter values: *B* = 5, α = 0.001, γ = 20, ρ = 0.7. Grey line corresponds to the initial amount of phage and values below this line indicate a lack of phage amplification.

The observed density-dependent phage amplification suggested that Acr-phages may cooperate in order to successfully amplify. For example, if co-infections were required to effectively suppress host resistance, epidemiological tipping points could correspond to parasite densities where co-infections become common (Regoes et al., 2002). However, this hypothesis is unlikely to explain our results because the tipping points occurred at MOI values where co-infections are expected to be rare (Figure 2). To explore what factors may cause the observed epidemiological tipping points at low MOIs and in the absence of phage evolution, we generated a theoretical model (see STAR Methods for a detailed description of the model and the differential equations). Given that we aimed to understand how the observed epidemiological tipping points could emerge without escape-phage evolution, we modeled CRISPR-phage population dynamics using fixed bacterial and phage genotypes. In the model, we can manipulate both the efficacy ρ of CRISPR resistance in the bacteria (ρ increases with the number of spacers targeting the phage) as well as the efficacy ϕ of Acr in the phage (consistent with the EOP data). In this form, the model predicts that upon infection of 10^6^ bacteria with CRISPR resistance, phages expressing a strong Acr can always amplify, regardless of the initial phage density (Figure S4, ϕ = 0.67, purple line), whereas phages with a weak Acr can never amplify (Figure S4, ϕ = 0.6 and ϕ = 0.5, magenta and green lines, respectively; gray lines correspond to the initial amount of phage, and values below this line indicate a lack of phage amplification). Given that these model predictions are inconsistent with our experimental data, we then extended the model by incorporating the assumption that during failed infections, some Acr proteins are produced that cause the surviving host to enter a “suppressed” state (S). This immunosuppression decreases the efficacy of host resistance and allows following phages to exploit these bacteria (Figure 4A). Crucially, if the immunosuppressed state is assumed, the model predicts epidemiological tipping points and, in accordance with our empirical data, these tipping points occur at MOIs far below 1 (Figure 4B). Besides, our experimental observations that the position of the tipping points shifts when Acrs are weaker or host resistance is stronger (Figure 2) are fully explained by our model when we vary the effect of the efficacy ϕ of the Acr in the phage (Figure 4B) or the efficacy ρ of CRISPR resistance in the bacteria (i.e., the number of spacers in the host targeting the phage; Figure 4C). Moreover, longer periods of immunosuppression shift the tipping points to lower phage densities, as it increases the probability that a host will be re-infected when it is still in the immunosuppressed state (Figure 4D, indicated by γ). This model therefore predicts that Acr-phage infections can cause bacteria with CRISPR resistance to become immunosuppressed, allowing cooperation between sequentially infecting Acr-phages to overcome CRISPR immunity, which is a critical factor in determining whether Acr-phages can amplify.

### Unsuccessful Infections by Acr-Phages Cause Hosts with CRISPR Resistance to Become Immunosuppressed

**Figure 5:**
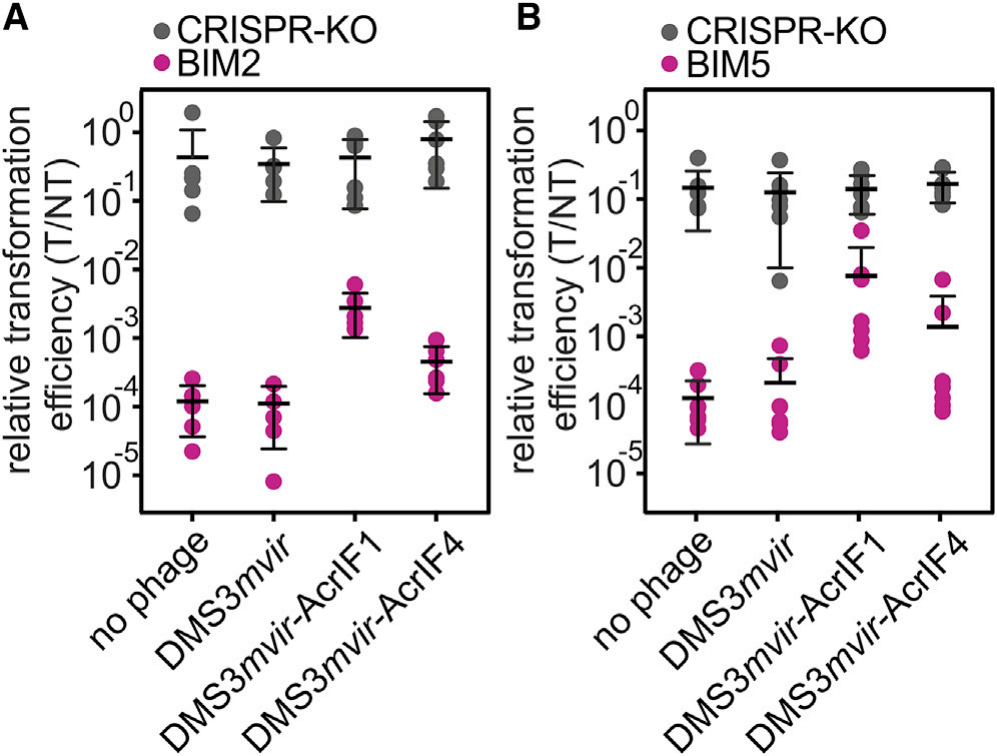
Unsuccessful Infections by Acr-Phages Cause Hosts with CRISPR Resistance to Become Immunosuppressed (A) Relative transformation efficiencies (RTE) of CRISPR-KO (gray data points) or BIM2 (purple data points) pre-infected with 1.6 × 10^9^ pfus of either DMS3*mvir*, DMS3*mvir*-AcrIF1, or DMS3*mvir*-AcrIF4 or not phage infected. Each data point represents an independent biological replicate (n = 6). (B) RTE of CRISPR-KO (gray data points) or BIM5 (purple data points) pre-infected as described for (A). Each data point represents an independent biological replicate (n = 7). In addition, we show the mean and 95% CI for each treatment. See also Figure S5 and Table S2.

To validate this model, we tested the key assumption that unsuccessful infections by Acr-phages cause hosts with CRISPR resistance to become immunosuppressed. To this end, we pre-infected BIM2 and BIM5 with Acr-phages at a low MOI (∼0.3) and subsequently washed away all remaining phages from the culture. We then measured the relative transformation efficiency (RTE) of the surviving cells by transforming pre-infected bacteria with either a CRISPR-targeted plasmid (T) or a non-targeted plasmid (NT). For all phage treatments, the RTE of pre-infected CRISPR-KO bacteria and no-phage controls were not significantly different, as expected (Figure 5; Table S2; F_1,51_ = 1.12, p = 0.35). However, when BIM2 or BIM5 were pre-infected with phage DMS3*mvir*-AcrIF1, the RTE increased significantly compared to the DMS3*mvir* and no-phage controls (Figure 5; Table S2; BIM2: F_1,17_ = 26.82, p < 0.0001; BIM5: F_1,20_ = 20.16, p < 0.0001), demonstrating lasting immunosuppression of hosts with CRISPR resistance following an unsuccessful infection with Acr-phages. Consistent with its weaker Acr activity, lasting immunosuppression following infection with DMS3*mvir*-AcrIF4 was only observed in BIM2 (Figure 5; Table S2; F_1,17_ = 5.26, p < 0.05) and not in BIM5 (F_1,20_ = 2.07, p = 0.15).

## Discussion

**Figure S5:**
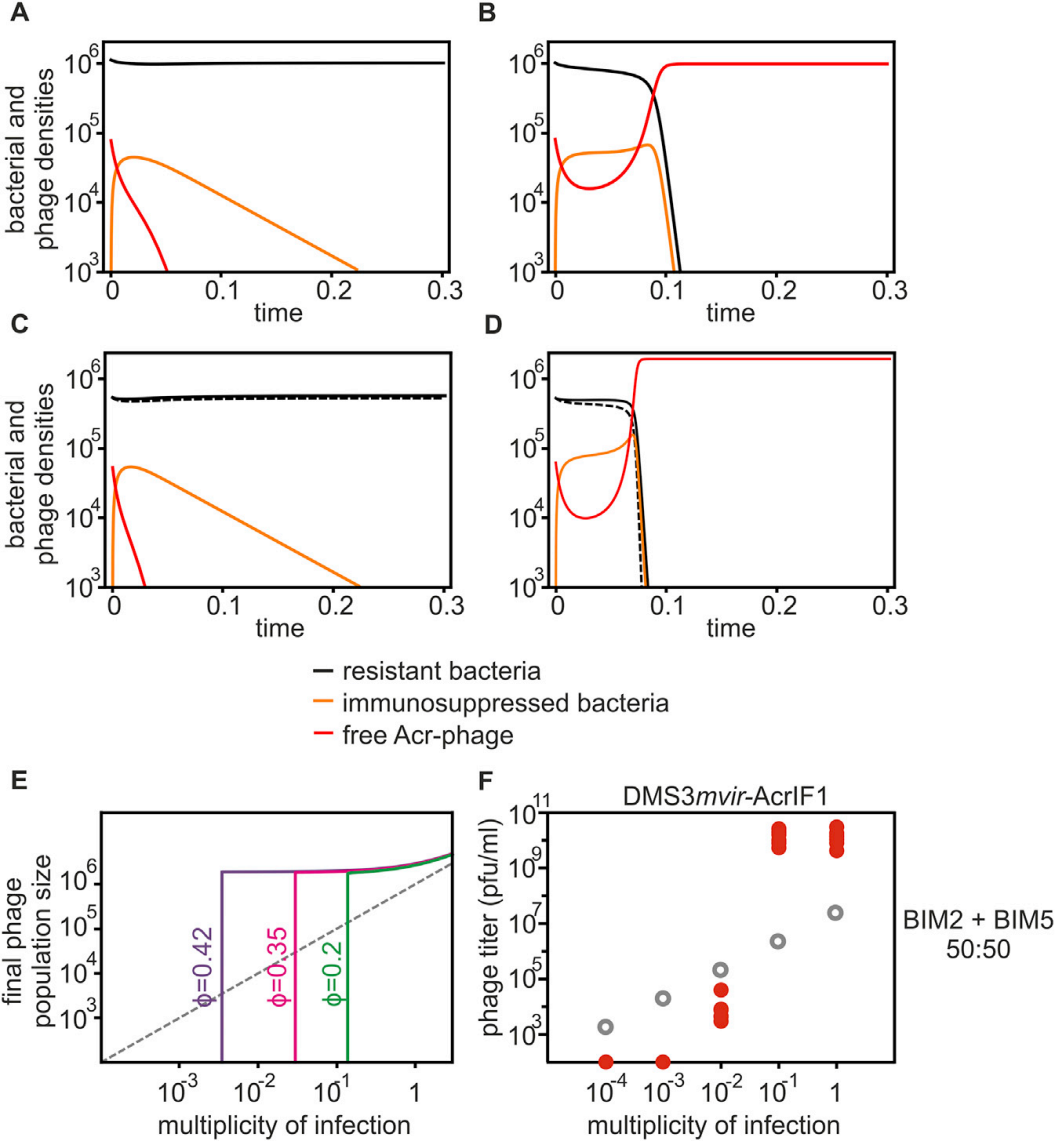
Model Predictions of the Temporal Population Dynamics of Acr-Phage and Resistant and Immunosuppressed Hosts, Related to Figure 5 (A and B) Model predictions for the densities of resistant bacteria (black), immunosuppressed bacteria (orange) and phages (red) across time for two initial inoculum sizes: (A) V(0) = 5.10^4^, (B) V(0) = 8.10^4^. Other parameter values: R(0) = 10^6^, *B* = 5, α = 0.001, γ = 20, ρ = 0.7. (C and D) Model predictions of the temporal population dynamics of Acr-phage and resistant and immunosuppressed hosts when the bacteria is initially composed of two different resistant hosts with different efficacy of resistance (*ρ*_*1*_ = 0.7 and *ρ*_*2*_ = 0.4) in equal frequency, with the densities of the two resistant bacteria (full and dashed black lines, respectively), immunosuppressed bacteria (orange) and phages (red) across time for two initial inoculum sizes: (C) V(0) = 5.10^4^, (D) V(0) = 6.10^4^. Other parameter values: *B* = 5, α = 0.001, γ = 20. (E) Effect of initial Acr-phage inoculum density on the phage density at 24 hpi for different values of Acr efficacy (ϕ = 0.42 (purple), ϕ = 0.35 (magenta) and ϕ = 0.2 (green)) when the bacteria is initially composed of two different resistant hosts with different efficacy of resistance (*ρ*_*1*_ = 0.7 and *ρ*_*2*_ = 0.4) in equal frequency. Other parameter values: *B* = 5, α = 0.001, γ = 20. (F) Viral titers at 24 hpi of a 50:50 mix of BIM2 and BIM5 with 10^4^, 10^5^, 10^6^, 10^7^ or 10^8^ pfus DMS3*mvir*-AcrIF1. Grey circles indicate the phage titers (pfu/ml) at the start of the experiment. Colored points represent phage titers at 24 hpi; each data point represents an independent biological replicate (n = 6). The limit of detection is 200 pfu/ml.

The discovery of *acr* genes has been a major breakthrough in CRISPR-Cas research (Bondy-Denomy et al., 2013). Much progress has been made toward biochemical characterization of Acrs and the unraveling of their molecular mode of action. Here, we studied the population dynamics associated with CRISPR-Acr interactions and demonstrated that the initial density of Acr-phages that infect bacteria with CRISPR resistance determines whether phages go extinct or amplify. Our data and theory offer a parsimonious explanation for these epidemiological tipping points based on long-term suppression of CRISPR resistance following an unsuccessful infection, which is consistent with the slow dissociation kinetics of Acr-Cas protein complexes (Chowdhury et al., 2017). During the initial stages of infection, Acr-phage densities decline due to the high proportion of unsuccessful infections (Figures S5A and S5B). However, as the densities of immunosuppressed hosts increase, a greater proportion of infections becomes successful. If the initial densities of Acr-phages are high enough, densities of immunosuppressed hosts reach a critical threshold where the amount of new Acr-phages that are produced from successful infections outweighs the loss of Acr-phages due to unsuccessful infections, causing the epidemic to take off (Figure S5B). If this critical threshold is not reached, the Acr-phages go extinct, and immunosuppressed hosts revert to their resistant state (Figure S5A).

We ruled out that the need for high Acr-phage densities is simply linked to the evolution of phage escape mutations. When phages lack *acr* genes, escape mutations are known to be an important determinant for phage persistence in the face of CRISPR-resistant bacteria (Deveau et al., 2008, Semenova et al., 2011, Weinberger et al., 2012, Paez-Espino et al., 2013, Paez-Espino et al., 2015), provided the CRISPR-resistant clones carry a single spacer (Levin et al., 2013) and the diversity of single spacer clones in the bacterial population is low (Iranzo et al., 2013, Childs et al., 2014, van Houte et al., 2016b). While escape mutations in Acr-phages were not required for phage amplification at high phage densities, they were still found to emerge when the Acr was weak and the host carried only one spacer. Evolution of escape mutants may therefore contribute to Acr-phage persistence in nature where phages exist in populations that are often genetically more heterogeneous than the initially clonal populations used in our experiments.

When phages overcome CRISPR resistance, natural selection likely favors the evolution of alternative host defense mechanisms (van Houte et al., 2016a). Indeed, we observed that bacteria carrying two spacers targeting phage DMS3*vir*-AcrIF1 evolved surface-modification-based resistance at 2 dpi when exposed to high Acr-phage densities but retained their CRISPR-resistance phenotype when exposed to low Acr-phage densities (Figure S3). As expected, this was associated with a decreased fitness of the bacteria with CRISPR resistance relative to those with surface-modification-mediated resistance at high but not at low Acr-phage densities (Figure S3). In nature, bacteria inevitably have greater access to genes encoding for alternative defense strategies that may be selected instead of CRISPR-Cas or in combination with CRISPR-Cas to provide synergistic levels of resistance, as was found to be the case for CRISPR-Cas and restriction-modification (Hynes et al., 2014). Similarly, phage-encoded *acr* genes may drive the evolution of multiple CRISPR subtypes being encoded by the same host to provide functional redundancy, explaining why hosts often need a diversified defense arsenal.

The artificial lab media used in our experiments inevitably lacks much of the biotic and abiotic complexity found in natural environments. For example, in natural environments, different CRISPR genotypes often coexist (Andersson and Banfield 2008), whereas in our experiments, we studied clonal populations of bacteria with CRISPR resistance. However, our theoretical and experimental analyses suggest that the coexistence of different CRISPR genotypes does not impact the observed epidemiological dynamics of Acr-phages (Figures S5C–S5F). Metagenomics analyses of bacteria-phage interactions in natural communities will be essential to further map the evolutionary and population dynamics of Acr-phages and their bacterial hosts.

Our experimental data suggest that the long-term immunosuppression following a failed infection by Acr-phages is an important factor in determining Acr-phage population dynamics and is cooperative in that it provides a benefit to Acr-phages that sequentially infect the same host to overcome CRISPR resistance. Future studies aimed at measuring the costs and benefits associated with phage-encoded *acr* genes and their natural ecology will be critical to understanding the evolutionary drivers of Acr-phage cooperation. Specifically, it is unclear whether lasting immunosuppression evolved because of the indirect fitness benefits associated with the enhanced infection success of clone mates in the population or whether it is primarily a by-product of the direct individual-level benefits of suppressing the host immune system (Sachs et al., 2004, West et al., 2007).

Positive density-dependent fitness effects, such as the one observed here for Acr-phages, play an important role in various ecological contexts, such as species invasions, extinctions, and disease epidemics (Courchamp et al., 1999, Stephens and Sutherland, 1999). Existing theory predicts that parasite density-dependent tipping points in disease epidemics can occur when the infection dynamics of an individual host depends on the parasite dose, for example when there is a threshold in the number of co-infecting parasites that are required to establish a successful infection (Regoes et al., 2002). This work shows that epidemiological tipping points can also take place under conditions where parasite densities are too low for co-infections to be common if unsuccessful infections leave behind an immunosuppressed host. The profound epidemiological consequences that were found to be associated with lasting immunosuppression in our empirical system warrant future studies to explore whether similar effects play a role in the epidemiology of other infectious diseases.

## STAR★Methods

### Key Resources Table

**Table:** 

REAGENT or RESOURCE	SOURCE	IDENTIFIER
**Bacterial and Virus Strains**		

*P. aeruginosa* UCBPP-PA14	O’Toole Lab, Cady et al., 2012	NCBI: NC_008463.1, Table S3
*P. aeruginosa* UCBPP-PA14 *csy3::LacZ*	O’Toole Lab, Cady et al., 2012	N/A
*P. aeruginosa* UCBPP-PA14 BIM2	Westra Lab, Westra et al., 2015	Table S3
*P. aeruginosa* UCBPP-PA14 BIM3	Westra Lab, This study	Table S3
*P. aeruginosa* UCBPP-PA14 BIM4	Westra Lab, This study	Table S3
*P. aeruginosa* UCBPP-PA14 BIM5	Westra Lab, This study	Table S3
*P. aeruginosa* UCBPP-PA14 *csy3::LacZ* surface mutant 1	Westra Lab, Westra et al., 2015	N/A
DMS3*vir*	O’Toole Lab, Cady et al., 2012	N/A
DMS3*mvir*	O’Toole Lab, Cady et al., 2012	https://github.com/s-meaden/landsberger/blob/master/DMS3mvir.gff.gz
DMS3*vir*-AcrIF1	Bondy-Denomy lab, van Houte et al., 2016b	N/A
DMS3*mvir*-AcrIF1	Davidson lab, Bondy-Denomy et al., 2013	https://github.com/s-meaden/landsberger/blob/master/DMS3mvirAcrF1.gff.gz
DMS3*mvir*-AcrIF4	Bondy-Denomy Lab, This study	https://github.com/s-meaden/landsberger/blob/master/DMS3mvirAcrF4.gff.gz
JBD26	Davidson lab, Bondy-Denomy et al., 2013	NCBI: JN811560.1
JBD30	Davidson lab, Bondy-Denomy et al., 2013	NCBI: JX434032.1

**Chemicals, Peptides, and Recombinant Proteins**		

Agar	VWR	Cat#A10752
LB broth	Fisher	Cat#12891650
LB agar	Fisher	Cat#10734724
Sodium chloride	Fisher	Cat#15505945
Di-Sodium hydrogen phosphate heptahydrate	VWR	Cat#1.06575.1000
Potassium phosphate monobasic	Sigma-Aldrich	Cat#P9791
Ammonium chloride	Sigma-Aldrich	Cat#A9434
Magnesium sulfate heptahydrate	VWR	Cat#25163.290
Calcium chloride	Sigma-Aldrich	Cat#C1016
D-(+)-Glucose	Sigma-Aldrich	Cat# G7021
X-Gal (5-Bromo-4-chloro-3-indolyl-β-D-galactopyranoside)	VWR	Cat# 437132J
Chloroform	Fisher	Cat# 10727024
Sucrose	Fisher	Cat# 10346150
Gentamicin sulfate	VWR	Cat# A1492
L-(+)-Arabinose	Sigma-Aldrich	Cat#A3256
HindIII-HF	NEB	Cat#R3104
T4 DNA Ligase	NEB	Cat# M0202
T4 Polynucleotide Kinase	NEB	Cat# M0201
Antarctic Phosphatase	NEB	Cat# M0289
RedSafe DNA Stain	Chembio	Cat#21141
TAE Buffer	Fisher	Cat#10542985
Agarose	Bioline	Cat#BIO-41025

**Critical Commercial Assays**		

Phage DNA Isolation Kit – 46850	Geneflow	Cat# P4-0135
Thermo Scientific GeneJET Plasmid Miniprep Kit	Fisher	Cat# 15563561 (K0502)
QIAquick Gel Extraction Kit	QIAGEN	Cat# 28704

**Deposited Data**		

Sequence data	European Nucleotide Archive	ENA: PRJEB25016
Raw data	Mendeley Data	https://doi.org/10.17632/vt434wb4b4.1

**Oligonucleotides**		

CRsp1FW_HindIII: AGCTTACCGCGCTCGA CTACTACAACGTCCGGCTGATGGA	IDT	N/A
CRsp1Rv_HindIII: AGCTTCCATCAGCCG GACGTTGTAGTAGTCGAGCGCGGTA	IDT	N/A
Primer 7: CTAAGCCTTGTACGAAGTCTC	IDT	N/A
Primer 8: CGCCGAAGGCCAGCGCGCCGGTG	IDT	N/A
Primer 10: GCCGTCCAGAAGTCACCACCCG	IDT	N/A
Primer 11: TCAGCAAGTTACGAGACCTCG	IDT	N/A

**Recombinant DNA**		

pHERD30T	Davidson lab, Bondy-Denomy et al., 2013	GenBank: EU603326.1
pHERD30T*targ*	Westra Lab, This study	N/A

**Software and Algorithms**		

Cutadapt version 1.2.1	Martin, 2011	https://github.com/marcelm/cutadapt
Sickle version 1.200	Joshi and Fass, 2011	https://github.com/najoshi/sickle
Flash version 1.2.11	Magoč and Salzberg 2011	https://ccb.jhu.edu/software/FLASH/
Bwa mem version 0.7.12	Li and Durbin, 2009	http://bio-bwa.sourceforge.net/
Samtools mpileup version 0.1.19	Li et al., 2009	http://samtools.sourceforge.net/
SPAdes version 3.5.0	Bankevich et al., 2012	http://bioinf.spbau.ru/en/content/spades-download-0
Bandage version 0.8.1	Wick et al., 2015	https://rrwick.github.io/Bandage/
Prokka version 1.12	Seemann, 2014	https://github.com/tseemann/prokka
R version 3.4.1	R Core Team, 2014	https://www.R-project.org/
Sequence analysis script	This paper	https://github.com/s-meaden/landsberger
Mathematica version 11.2	Wolfram	https://www.wolfram.com/mathematica/quick-revision-history.html
SigmaPlot version 12	Systat Software	http://www.sigmaplot.co.uk/products/sigmaplot/sigmaplot-details.php
CorelDRAW version x7	Corel	https://www.coreldraw.com/en/product/graphic-design-software/?topNav=en

**Other**		

Illumina sequencing	MiSeq platform	https://emea.illumina.com/systems/sequencing-platforms/miseq/specifications.html

### Contact for Reagent and Resource Sharing

Further information and requests for resources and reagents should be directed to and will be fulfilled by the Lead Contact, Edze R. Westra (westra.edze@gmail.com).

### Experimental Model and Subject Details

#### Bacterial strains

*P. aeruginosa* UCBPP-PA14 (referred to as WT, carrying one spacer targeting DMS3*mvir,* see Table S3), and derived *P. aeruginosa* UCBPP-PA14 strains BIM2, BIM3, BIM4, and BIM5, and the strain *P. aeruginosa* UCBPP-PA14 *csy3::LacZ* (referred to as CRISPR-KO, since it carries a disruption of an essential *cas* gene that causes the CRISPR-Cas system to be non-functional), and the *P. aeruginosa* UCBPP-PA14 *csy3::LacZ*-derived surface mutant 1 (referred to as SM (Westra et al., 2015)) were used in all experiments. Phage was amplified on *P. aeruginosa* UCBPP-PA14 *csy3::LacZ*. Cells were grown overnight at 37°C in either LB broth, or M9 medium (22 mM Na_2_HPO_4_; 22 mM KH_2_PO_4_; 8.6 mM NaCl; 20 mM NH_4_Cl; 1 mM MgSO_4_; 0.1 mM CaCl_2_) supplemented with 0.2% glucose.

#### Virus strains

Temperate phages JBD26 and JBD30 were used in efficiency of plaquing experiments and infection assays in liquid medium and have been previously described (Bondy-Denomy et al., 2013). Recombinant lytic phages DMS3*vir*, DMS3*vir*-AcrIF1, DMS3*mvir*, DMS3*mvir*-AcrIF1 and DMS3*mvir*-AcrIF4 have been used in all experiments (described in Cady et al., 2012, Bondy-Denomy et al., 2013, and van Houte et al., 2016b).

### Method Details

#### Evolution of P. aeruginosa PA14-derived BIMs

The *P. aeruginosa* UCBPP-PA14 BIM2 strain (referred to as BIM2, carrying two spacers targeting DMS3*mvir*, see Table S3) was evolved during infection of *P. aeruginosa* UCBPP-PA14 with DMS3*vir* (Westra et al., 2015). *P. aeruginosa* UCBPP-PA14 BIM3 (referred to as BIM3, carrying three spacers targeting DMS3*mvir*), BIM4 (referred to as BIM4, carrying one newly acquired and two pre-existing spacers targeting JBD26 and JBD30) and BIM5 strains (referred to as BIM5, carrying five spacers targeting DMS3*mvir*) were generated by challenging *P. aeruginosa* UCBPP-PA14 BIM2 bacteria with escape phage in multiple rounds, giving rise to BIM3, and finally BIM4 and BIM5.

#### Generation of recombinant phage

DMS3*mvir*-AcrIF4 phage was generated by homologous recombination with a plasmid-encoded *acrIF4* gene (flanked by homology arms) to replace the native *acrIE3* gene of DMS3, as described in Bondy-Denomy et al., 2013, followed by truncation of the c-repressor gene as described in Cady et al., 2012.

#### Efficiency of Plaquing assays

Efficiency of plaquing (EOP) assays were carried out on square polypropylene plates containing LB with 1.5% agar. A mixture of molten soft LB agar (0.5%) and 300 μL of bacteria (grown overnight in M9 medium supplemented with 0.2% glucose) were poured on top of the hard agar layer. Next, 5 μL of serially diluted phage was spotted on the resulting plates, which were subsequently incubated overnight at 37°C and plaques were enumerated the next day.

#### Competition assays to measure fitness

Competition experiments were performed in glass vials in 6 mL M9 medium supplemented with 0.2% glucose. Competition experiments were initiated by inoculating 1:100 from a 1:1 mixture of overnight cultures (grown in M9 medium + 0.2% glucose) of the strain with CRISPR resistance and either the sensitive CRISPR-KO strain or a CRISPR KO-derived surface mutant (described in Westra et al., 2015). For the competitions between the strains with CRISPR resistance (WT, BIM2 or BIM5) and the sensitive CRISPR-KO strain 10^4^ plaque forming units (pfus) of either DMS3*mvir*-AcrIF1 or DMS3*mvir*-AcrIF4 phage was added to each glass vial. At 0 and 24 hours after the start of the competition experiment samples were taken and cells were serially diluted in M9 salts (22 mM Na_2_HPO_4_; 22 mM KH_2_PO_4_; 8.6 mM NaCl; 20 mM NH_4_Cl) and plated on LB agar supplemented with 50 μg⋅ml^-1^ X-gal (to allow discrimination between bacteria with CRISPR resistance (white) and sensitive CRISPR-KO (blue) bacteria). For all competitions, a control competition experiment was performed in the absence of phage. For the competitions between bacteria with CRISPR resistance (BIM3) and surface mutants, 10^4^, 10^5^ or 10^6^ pfus of DMS3*vir*-AcrIF1 phage was added to each glass vial. At 0 and 48 hours after the start of the competition experiment samples were taken and processed as above.

#### Analysis of bacterial evolution of resistance

For evolution of bacterial resistance experiments 6 mL of M9 medium supplemented with 0.2% glucose were inoculated with approximately 10^8^ bacteria, and 600 mL was inoculated with approximately 10^10^ bacteria, from fresh overnight cultures of BIM3 (2 spacers targeting DMS3*vir*-AcrIF1 phage). These cultures were infected with 10^4^, 10^5^, 10^6^, 10^7^, and 10^8^ pfu of DMS3*vir*-AcrIF1 (Figure S3A) or 10^6^ pfu of DMS3*vir*-AcrIF1 (Figure S3C), followed by incubation at 37°C and shaking at 180 rounds per minute (rpm). Cultures were transferred 1:100 into fresh medium every 24 hours. Evolution of resistance was determined after 48 h by streaking individual clones through DMS3*vir* and DMS3*vir*-AcrIF1. Surface modification was confirmed by colony morphology, broad-range resistance of colonies to DMS3*vir* phages carrying Acr genes, and lack of newly acquired spacers. CRISPR-Cas-mediated immunity was confirmed by PCR using primers 5′-CTAAGCCTTGTACGAAGTCTC-3′ and 5′-CGCCGAAGGCCAGCGCGCCGGTG-3′ for CRISPR array 1, and primers 5′-GCCGTCCAGAAGTCACCACCCG-3′ and 5′-TCAGCAAGTTACGAGACCTCG-3′ for CRISPR array 2.

#### Infection assays in liquid medium

Infection assays were performed in glass vials by inoculating 6 mL M9 medium supplemented with 0.2% glucose with approximately 10^8^ colony forming units (cfus) bacteria from fresh overnight cultures (also grown in M9 medium + 0.2% glucose) of either the CRISPR-KO, WT, BIM2 or BIM5 strain. To these microcosms 10^4^, 10^5^, 10^6^, 10^7^ or 10^8^ pfus of either DMS3*mvir*, DMS3*mvir*-AcrIF1 or DMS3*mvir*-AcrIF4 phage were added. Microcosms were incubated at 37°C while shaking at 180 rpm. Phage was extracted at 24 hours after the start of the experiment by chloroform extraction on all samples (sample: chloroform 10:1 v/v), and phage titers were determined by spotting isolated phage samples on a lawn of CRISPR-KO bacteria. For the experiment shown in Figures S2C–S2F and S5F the same protocol was used but with 10^8^ pfus DMS3*mvir* added to each treatment at the start of the experiment (Figures S2D and S2F) or using a bacterial population that consisted of an equal mix of BIM2 and BIM5 (Figure S5F). For the infection assays in which phage densities were manipulated by using different volumes of growth medium (Figure 3), glass vials containing 6 mL M9 medium supplemented with 0.2% glucose were used for the high phage-density (HPD) treatment. These were inoculated with approximately 10^8^ cfus of bacteria from fresh overnight cultures of the CRISPR-KO, BIM2 or BIM5 strains, as above. For the low phage-density (LPD) treatment Duran 1L glass bottles containing 600 mL M9 medium supplemented with 0.2% glucose were used, which were inoculated with approximately 10^10^ cfus of bacteria from the same overnight cultures. To both glass vials and bottles phage was added (10^6^ pfus of DMS3*mvir*-AcrIF1 or DMS3*mvir* for infection of BIM2; 10^7^ pfus of DMS3*mvir*-AcrIF1 or DMS3*mvir* for infection of BIM5; 10^8^ pfus of DMS3*mvir*-AcrIF4 or DMS3*mvir* for infection of both BIM2 and BIM5). Glass vials and bottles were incubated at 37°C while shaking at 180 rpm. Total phage was extracted at 24 hours after the start of the experiment and phage titers were determined as described above.

#### Deep sequencing of phages

From the experiments shown in Figures 2D–2F, 2H, 2I, 2K, and 2L, phage was isolated at 24 hpi from the treatments that were infected with 10^8^ pfus of phage (MOI = 1). These phages were used for a new round of infection assays in liquid media (Figure S2B), effectively following the methods described above, and for deep sequencing analysis (Figure S2A). To obtain sufficient material for the latter, isolated phage was amplified on plate by infecting a lawn of the CRISPR-KO strain and harvesting phage by adding M9 salts solution and subsequent chloroform extraction. Phage samples from all replicates within a single treatment were pooled. As controls, ancestral DMS3*mvir*, DMS3*mvir*-AcrIF1 and DMS3*mvir*-AcrIF4 phage were processed in parallel. Phage genomic DNA extraction was performed with 600 μL sample at approximately 10^12^ pfu/ml using the Norgen phage DNA isolation kit, following the manufacturer’s instructions. Barcoded Illumina Truseq Nano libraries were constructed from each DNA sample with an approximately 350 bp insert size and 2 × 250 bp reads generated on an Illumina MiSeq platform. Reads were trimmed using Cutadapt version 1.2.1 and Sickle version 1.200 and then overlapping reads merged using Flash version 1.2.11 to create high quality sequence at approximately 8000 × coverage of DMS3*mvir* per sample. These reads were mapped to the DMS3 reference genome (NCBI RefSeq: NC_008717) using bwa mem version 0.7.12 and allele frequencies of single nucleotide polymorphisms within viral target regions quantified using samtools mpileup version 0.1.19. Draft genomes were constructed using the ancestral sample sequences which were randomly subsampled to 10000 reads per sample. Assembly was done using SPAdes version 3.5.0 run in ‘careful’ mode. Assemblies were visualized using Bandage version 0.8.1 and a single contig was extracted based on coverage. Contigs were annotated using Prokka version 1.12.

#### Long-term immunosuppression experiment

For the long-term immunosuppression experiment (Figure 5) bacteria were transformed with plasmid pHERD30T and a pHERD30T-derived plasmid that was *a priori* targeted by the PA14 WT Type I-F CRISPR-Cas system. This mutant plasmid pHERD30T*targ* was generated by inserting a 32-nt protospacer sequence flanked by a GG protospacer adjacent motif (PAM) with full complementarity to spacer 1 in CRISPR locus 2 of the PA14 WT Type I-F CRISPR-Cas system. This was done by ligation of T4 Polynucleotide Kinase phosphorylated oligonucleotides that upon annealing create overhangs that are compatible with *Hind*III ligation (5′-agcttACCGCGCTCGACTACTACAACGTCCGGCTGATGGa-3′ and 5′-agcttCCATCAGCCGGACGTTGTAGTAGTCGAGCGCGGTa-3′, *Hind*III overhangs in small caps, protospacer sequence in capitals and PAM underlined) in the *Hind*III-digested Antarctic phosphatase-treated pHERD30T vector. Suppression of CRISPR immunity by Acr was measured through a transformation assay. Overnight cultures of CRISPR-KO, BIM2 or BIM5 bacteria (approximately 5^∗^10^9^ cfus) grown in LB broth were either not infected or infected with 1.6^∗^10^9^ pfus DMS3*mvir*, DMS3*mvir*-AcrIF1 or DMS3*mvir*-AcrIF4 in 50 mL Falcon tubes containing 10 mL of Luria-Bertani (LB) broth, and incubated at 37°C while shaking at 180 rpm for 2 hours. Bacteria were harvested by spinning at 3500 rpm for 30 minutes. A sample from the supernatant was used to quantify phage titers following chloroform extraction as described above (data not shown). The bacterial pellet was further processed for the transformation assay. First, cells were washed twice in 1 mL of a 300 mM sucrose solution to make them competent (Choi et al., 2006). Next, the pellet was resuspended in 300 μL of 300 mM sucrose, and 100 μL from this was used for plating on LB agar to enumerate total bacterial cfus after the infection and sucrose-washing steps (data not shown). Finally, the remaining 200 μL was divided in equal volumes over two eppendorf tubes, and used for electrotransformation with either plasmid pHERD30T (not targeted by CRISPR-Cas; NT) or pHERD30T*targ* (targeted by CRISPR-Cas; T). Electroporated bacteria were resuspended in 1 mL LB broth and incubated 1h at 37°C at 180 rpm. Bacteria were then pelleted and resuspended in 100 μL LB and plated on LB agar plates containing Gentamycin (50 μg⋅ml^-1^) and incubated for 16h at 37°C to allow transformants to grow.

#### Mathematical modeling

We developed the following epidemiological model to understand the dynamics of bacteriophages that carry an anti-CRISPR mechanism (Acr) in a host population with CRISPR resistance, with all simulations performed in Mathematica version 11.2.

Resistant bacteria may either be in their normal resistant state (the density of these bacteria is noted R(t)) or in an immunosuppressed state (the density of these bacteria is noted S(t)).

Initially the host population is homogeneous, consisting exclusively of bacteria with CRISPR resistance, and the density of these resistant bacteria is R(0). Then an inoculum of free Acr-phage particles with density V(0) is introduced in the host population. Free Acr-phage particles adsorb to the bacteria at a rate a. When a free Acr-phage adsorbs to a bacterium with CRISPR resistance three outcomes are possible:

(i) with probability ρ the Acr-phage genome is destroyed and there is no change in bacterial resistance (i.e., no immunosuppression). Hence, ρ is a measure of bacterial resistance and increases with the number of spacers targeting the phage,
(ii) with probability (1−ρ)ϕ the Acr-phage genome is not destroyed because of Acr activity. In this case the infection therefore leads to cell lysis, with the release of B new Acr-phage particles. The efficacy of Acr activity is thus measured with ϕ (the greater ϕ the higher the Acr efficacy is).
(iii) with probability (1−ρ)(1−ϕ), the Acr-phage fails to complete its lytic cycle but expresses some Acr before the Acr-phage genome is cleaved, which blocks bacterial CRISPR resistance causing the bacterium to become immunosuppressed. This state is reversible and immunosuppressed bacteria become resistant again at rate γ.

If an immunosuppressed bacterium is infected by an Acr-phage, the absence of immunity allows the Acr-phage to complete its lytic life cycle. This yields the following set of ordinary differential equations (see Figure 4A):

$$\dot{R}\left(t\right)=-\left(a\left(1-\rho \right)V\left(t\right)\right)R\left(t\right)+\gamma \;S\left(t\right)$$

$$\dot{S}\left(t\right)=a\left(1-\rho \right)\left(1-\phi \right)V\left(t\right)R\left(t\right)-\left(\gamma +a\;V\left(t\right)\right)S\left(t\right)$$

(1) $$\dot{V}\left(t\right)=a\left(1-\rho \right)\phi B\;V\left(t\right)R\left(t\right)+aB\;V\left(t\right)S\left(t\right)-a\left(S\left(t\right)+R\left(t\right)\right)V\left(t\right)$$

Initially there are no immunosuppressed bacteria around (i.e., S(0)=0) and if a few Acr-phage particles are introduced the Acr-phage population will increase if the Acr activity is higher than a threshold value:

(2) $$\phi >\phi_{0}=\frac{1}{\left(1-\rho \right)B}$$

In other words, when ϕ is low and ρ is high the phage population cannot take off when the inoculum of the Acr-phage is small (Figures 4B and 4C).

Yet, if one introduces a sufficiently high density of Acr-phages they will shut down the immunity of the bacterial population and will increase the density of immunosuppressed bacteria. At time T the following condition may be verified

(3) $$\phi >\phi_{0}\left(1-\frac{S\left(T\right)}{R\left(T\right)}\left(B-1\right)\right)$$

and the Acr-phage population may thus take off for lower values of Acr activity. Conditions (2) and (3) indicate a range of values of ϕ where the epidemiological outcome depends on the initial density of the Acr-phage (epidemiological bistability). Note that the threshold value of the density of Acr-phage leading to an epidemic depends also on the duration of the immunosuppression (see Figure 4D).

In Figure S4 we illustrate the epidemiological dynamics taking place when the initial density of the Acr-phage is either below or above the threshold value of the phage inoculum. Below the threshold (Figure S4A), the density of immunosuppressed bacteria does not reach a high enough level to satisfy condition (3) and the Acr-phage population goes extinct. Above the threshold (Figure S4B), the immunosuppressed bacteria reaches a high enough density, condition (3) is verified and the Acr-phage population can exploit the whole bacteria population.

The above model assumes that the host population is initially monomorphic (i.e., only one type of resistant bacteria). We can relax this assumption and consider a case where the initial population is composed of n different resistant bacteria. The initial frequency of these different bacteria is assumed to be $p_{i}$ and each resistant may have a different efficacy of resistance $\rho_{i}$. In this case, the threshold value of the Acr efficacy becomes (compare with (2)):

(4) $$\phi >\phi_{0}=\frac{1}{\sum_{i=1}^{n}p_{i}\left(1-\rho_{i}\right)B}$$

Below this threshold, the Acr-phage will be able to induce an epidemic only if the initial density of the virus is above a threshold density. In other words, the initial diversity of the host population does not affect qualitatively the behavior of the system: the existence of an epidemiological bistability is robust to the existence of an initially diverse bacteria population. In Figures S5C and S5D we illustrate the epidemiological dynamics taking place when the initial density of the Acr-phage is either below or above the threshold value of the phage inoculum in a situation where the initial bacterial population is polymorphic (compare with Figures S5A and S5B). In Figure S5E we show the effect of increasing inoculum size on the final phage population size in a situation where the initial bacterial population is polymorphic (compare with Figure 4B).

### Quantification and Statistical Analysis

All experiments were carried out in at least six biological replicates (n ≥ 6). Statistical parameters and tests are stated in figure legends, figures and results. Unless otherwise stated, the statistical tests used were general linear models with the appropriate error structure with significance determined by stepwise model simplification comparing full and null models using ANOVA. Model residuals were checked to satisfy the assumptions made by each model. In all cases the threshold for significance was p < 0.05. Analysis was conducted in R version 3.4.1. Figures were created using SigmaPlot version 12, and CorelDRAW version x7.

#### Efficiency of Plaquing assays

EOP was calculated based on the number of phage plaques on CRISPR-KO and CRISPR resistant hosts.

$$EOP=\frac{\#plaques\;on\;CRISPR\;resistant\;host}{\#plaques\;on\;CRISPR\;KO\;host}$$

#### Competition assays to measure fitness

Relative frequencies of the strains with CRISPR resistance compared to the competing CRISPR-KO strain were determined through colony numbers and used to calculate the relative fitness.

$$Relative\;fitness=\frac{\left(fraction\;strain\;A\;at\;t=x\right)\times \left(1-\left(fraction\;strain\;A\;at\;t=0\right)\right)}{\left(fraction\;strain\;A\;at\;t=0\right)\times \left(1-\left(fraction\;strain\;A\;at\;t=x\right)\right)}$$

#### Long-term immunosuppression experiment

Relative transformation efficiency was calculated using the number of transformed colonies on antibiotic selection plates for each of the four phage treatments.

$$Relative\;transformation\;efficiency=\frac{\#colonies\;transformed\;with\;T\;plasmid}{\#colonies\;transformed\;with\;NT\;plasmid}$$

### Data and Software Availability

Sequence analysis scripts and draft genome assemblies of DMS*mvir*, DMS3*mvir*-AcrIF1, and DMS3*mvir*-AcrIF4 can be accessed from https://github.com/s-meaden/landsberger under https://github.com/s-meaden/landsberger/blob/master/DMS3mvir.gff.gz, https://github.com/s-meaden/landsberger/blob/master/DMS3mvirAcrF1.gff.gz, and https://github.com/s-meaden/landsberger/blob/master/DMS3mvirAcrF4.gff.gz. Sequence data have been deposited in the European Nucleotide Archive under accession number ENA: PRJEB25016. Raw data have been deposited at Mendeley Data under https://doi.org/10.17632/vt434wb4b4.1.
