# Supplementary material for: Anti-CRISPR Phages Cooperate to Overcome CRISPR-Cas Immunity
Source: Cell. 2018 Aug 9;174(4):908–916.e12. doi: 10.1016/j.cell.2018.05.058 (PMC6086933; doi:10.1016/j.cell.2018.05.058)
Supplement: Document S1. Tables S1–S3 [file mmc1.pdf]

Cell, Volume *174*

Supplemental Information

Anti-CRISPR Phages Cooperate

to Overcome CRISPR-Cas Immunity

Mariann Landsberger, Sylvain Gandon, Sean Meaden, Clare Rollie, Anne Chevallereau, Hélène Chabas, Angus Buckling, Edze R. Westra, and Stineke van Houte

Supplementary Table 1. Related to Figure 3. ANOVA results for the effect of density of fold amplification for each host and phage combination tested.

Fig. 3 Panel	Host	Phage	n	F	P value
A	CRISPR-KO	DMS3*mvir*-AcrIF1	12	136.1	<0.0001
A	BIM2	DMS3*mvir*-AcrIF1	12	11.1	<0.01
B	CRISPR-KO	DMS3*mvir*-AcrIF4	12	345.8	<0.0001
B	BIM2	DMS3*mvir*-AcrIF4	12	221.3	<0.0001
C	CRISPR-KO	DMS3*mvir*-AcrIF1	12	1365.1	<0.0001
C	BIM5	DMS3*mvir*-AcrIF1	12	104.7	<0.0001
D	CRISPR-KO	DMS3*mvir*-AcrIF4	12	1166.9	<0.0001
D	BIM5	DMS3*mvir*-AcrIF4	12	867.1	<0.0001

Supplementary Table 2. Related to Figure 5. Mean and 95% c.i. values of the relative transformation efficiencies shown in Figure 5 on CRISPR-KO strain and BIMs.

	**BIM2 (Figure 5A)**				**BIM5 (Figure 5B)**			
	CRISPR KO		BIM2		CRISPR KO		BIM5	
Pre-treatment	mean	95% ci	mean	95% ci	mean	95% ci	mean	95% ci
no phage	4.7 10^{-1}	5.7 10^{-1}	1.3 10^{-4}	7.2 10^{-5}	1.5 10^{-1}	8.7 10^{-2}	1.3 10^{-4}	7.5 10^{-5}
DMS3*mvir*	3.8 10^{-1}	2.2 10^{-1}	1.2 10^{-4}	7.5 10^{-5}	1.3 10^{-1}	9.0 10^{-2}	2.2 10^{-4}	2.0 10^{-4}
DMS3*mvir*-AcrIF1	4.7 10^{-1}	3.0 10^{-1}	3.0 10^{-3}	1.5 10^{-3}	1.5 10^{-1}	6.2 10^{-2}	7.9 10^{-3}	9.3 10^{-3}
DMS3*mvir*-AcrIF4	8.6 10^{-1}	5.6 10^{-1}	4.9 10^{-4}	2.6 10^{-4}	1.8 10^{-1}	6.2 10^{-2}	1.4 10^{-3}	1.9 10^{-3}

Supplementary Table 3. Related to STAR methods. Summary of spacer sequences in CRISPR-Cas resistant bacterial strains used in this study.

Bacterial strains with CRISPR resistance used in this study are listed along with their experimentally acquired spacers against phage DMS3(*m*)*vir*. Presence and absence of matching protospacers in phage genomes used in this study is indicated with "+" and "-", respectively. Note that the naturally occurring spacers 17 and 20 of CRISPR 2 of *P. aeruginosa* UCBPP-PA14 have a perfect match with JBD26 and JBD30. The effectiveness of these spacers may be lessened due to the leader-distal localisation of these spacers within the CRISPR array compared to most recently acquired spacers such as spacer 1 CRISPR 2 (McGinn and Marraffini, 2016). The naturally occurring spacer 1 of CRISPR 2 of *P. aeruginosa* UCBPP-PA14 has a perfect match with DMS3*mvir* and the derived mutants (Cady et al., 2012).

Bacteria strain	Newly acquired spacers	Targeting of spacers (+/-) and total number of protospacers (in parentheses)							
		DMS3*mvir* DMS3*mvir*-AcrIF1 DMS3*mvir*-AcrIF4		DMS3*vir* DMS3*vir*-AcrIF1 DMS3*vir*-AcrIF4		JBD26		JBD30	
P. aeruginosa UCBPP-PA14 **WT**	none		(1)		(0)		(2)		(2)
P. aeruginosa UCBPP-PA14 **BIM2**	AACGGCCGACGCTTCTGGGTCGTCGTGAAAGT	+	(2)	+	(1)	-	(2)	-	(2)
P. aeruginosa UCBPP-PA14 **BIM3**	AACGGCCGACGCTTCTGGGTCGTCGTGAAAGT	+	(3)	+	(2)	-	(2)	-	(2)
	TGGACAACGGCAGCGCGATTCCCCGCGTGACC	+		+		-		-	
P. aeruginosa UCBPP-PA14 **BIM4**	AACGGCCGACGCTTCTGGGTCGTCGTGAAAGT	+	(4)	+	(3)	-	(3)	-	(3)
	TGGACAACGGCAGCGCGATTCCCCGCGTGACC	+		+		-		-	
	GTGCCGGACGCGGGGATGGTGTTGTCGAAAAC	+		+		+		+	
P. aeruginosa UCBPP-PA14 **BIM5**	AACGGCCGACGCTTCTGGGTCGTCGTGAAAGT	+	(5)	+	(4)	-	(2)	-	(2)
	TGGACAACGGCAGCGCGATTCCCCGCGTGACC	+		+		-		-	
	AGGACCAGGTCCGGCTCGCCCTGGAGCGTCCA	+		+		-		-	
	TGAACCAATGCTGATCGAGGCCGACGGCCCTG	+		+		-		-	
